# Natural Product Discovery by Direct Analysis in Real Time Mass Spectrometry

**DOI:** 10.5702/massspectrometry.S0081

**Published:** 2020-01-11

**Authors:** Joanne Y. Yew

**Affiliations:** 1 Pacific Biosciences Research Center, University of Hawai‘i at Mānoa, 1993 East West Road, Honolulu, HI 96822, USA

**Keywords:** DART MS, ambient mass spectrometry, chemotaxonomy, chemical fingerprinting, metabolomics, pheromone

## Abstract

Direct analysis in real time mass spectrometry (DART MS) is one of the first ambient ionization methods to be introduced and commercialized. Analysis by DART MS requires minimal sample preparation, produces nearly instantaneous results, and provides detection over a broad range of compounds. These advantageous features are particularly well-suited for the inherent complexity of natural product analysis. This review highlights recent applications of DART MS for species identification by chemotaxonomy, chemical profiling, genetic screening, and chemical spatial analysis from plants, insects, microbes, and metabolites from living systems.

## INTRODUCTION

Direct analysis in real time (DART) was first introduced in 2005^1)^ and together with desorption electrospray ionization (DESI),^2,3)^ is considered to be a pioneering method of ambient mass spectrometry (MS). Ambient MS entails sample analysis at atmospheric pressure without the need for extensive preparation and extraction, pre-treatment, chemical dopants, or matrices. Numerous other forms of ambient MS have since been introduced and are reviewed in greater detail elsewhere.^4,5)^ Here, I discuss the unique features of DART MS that make it particularly amenable to natural product analysis and review recent applications to plants, animals, microbes, and metabolites in living systems.

### DART MS ionization mechanism

**Figure 1:**
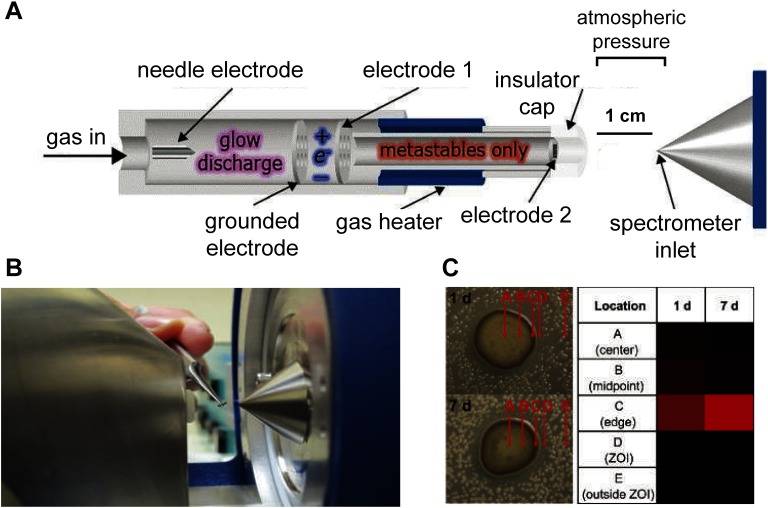
Fig. 1. Overview of DART ionization source and sample handling. (A) Schematic of DART source (courtesy of R. Cody, JEOL USA, Inc.). (B) Chemical profiling of a single fly. A single *Drosophila* (pomace fly) is held by forceps in the zone between the ionization source and the mass spectrometer inlet (picture courtesy of Y. N. Chiang, National University of Singapore). (C) A single *Leisingera* bacterial colony is sampled at 5 different locations over the course of 7 d. Subsequent analysis of the sampling probe by DART was able to track enrichment of the anti-bacterial product indigoidine at the outer edge of the colony.^11)^ (Figure reprinted with permission from M. Balunas and S. Nyholm, University of Connecticut.)

Ionization with DART occurs through a combination of Penning and chemical ionization at atmospheric pressure.^1,6)^ Within the DART ionization source, electrical discharge is applied to helium gas, generating a plasma (Fig. 1A). Cations, anions, and electrons are removed with grid electrodes leaving electronically excited neutral metastable species at the exit of the source. Samples are placed under ambient conditions in the zone between the ion source and the inlet of the mass spectrometer, a space which can be adjusted from 5–25 mm. Ion formation takes place *via* i) direct interaction of the analyte with helium atoms or ii) interactions with ions formed from secondary reactions of helium with water in the surrounding air. DART MS is best suited for the analysis of small molecules below *m*/*z* 1500. The temperature of the helium stream is adjustable from 50 to 550°C, a parameter that can be helpful for improving the thermal desorption of polar and heavier molecules.

### Specialized features of DART MS

DART ionization has several distinguishing features that are well-suited for natural product analysis. First, the open air configuration allows diverse sample types to be analyzed directly, including liquids, gases, living tissue, clothing, paper, thin layer chromatography (TLC) plates, and insect carcasses^7)^ (Fig. 1B). The chemical profile that is generated represents natural products as they are found in the context of the natural biological matrix. The minimal preparation significantly shortens analysis time, lessens the loss of material, and reduces artifacts from lengthy extraction and purification processes. Moreover, the sampling configuration allows chemical dopants to be readily introduced into the DART helium stream. For instance, trifluoroacetic acid or ammonia vapors placed in the ionization zone enhance the ionization of explosives^1)^ and triacylglycerols,^8)^ respectively. Pairing a directed ozone stream with DART ionization generates ozonolysis products from unsaturated fatty acids, allowing the carbon–carbon double bond position to be discerned.^9)^ By contrast, natural product analysis using conventional gas chromatography (GC) or liquid chromatography (LC) approaches requires multiple preparatory steps including extraction, chromatographic separation, pooling and concentration, and derivatization. Extensive processing can necessitate significant amounts of starting material in order to compensate for sample loss. In addition, degradation, oxidation, and other artifacts can occur during sample preparation. A second distinct advantage of DART MS is that polar and heavier molecules often missed by GCMS can be detected by DART MS. Last, the near-instantaneous profiling by DART MS allows chemical reactions or changes in the chemical profile of live organisms to be monitored over multiple time points^10–12)^ (Fig. 1C).

As with any analytical technique, DART ionization has several inherent limitations. First, fragmentation can occur at higher plasma temperatures, hindering spectra interpretation and accurate determination of the mass of intact molecules.^6)^ However, for some samples, this feature can be informative because structural information can be inferred from the *m*/*z* of decomposition fragments (see section below on plant tissue). A second limitation of DART ionization is that analytes are subject to oxidation artifacts, an occurrence that is dependent on the distance from the capillary outlet.^6)^ Lastly, saturated hydrocarbons can undergo hydride abstraction. In this scenario, signals from aliphatic hydrocarbons, detected as [M−H]^+^, are indistinguishable from signals corresponding to monounsaturated hydrocarbons of the same carbon length (detected as [M+H]^+^),^13)^ hindering quantitative analysis. A second method such as GCMS or deuterium exchange is needed to distinguish between the two compounds. Thus, complementing DART MS with other analytical methods is critical especially when measuring uncharacterized natural products for the first time.

Despite these drawbacks, DART MS is a powerful analytical method for the targeted analysis of small molecules from natural substrates and for chemical fingerprinting, an application which compares the overall pattern of signals generated from an analyte and does not necessitate the identification of individual components. Below, I provide recent applications of DART MS for the analysis of plants, animals, microbes, and metabolites from living tissue and biological fluids.

## PLANT TISSUE

### Chemotaxonomy by lipids, alkaloids, and saccharides

Species identification of organisms is most commonly performed using morphological features and/or DNA barcoding. The latter uses a short DNA nucleotide sequence that is searched against a reference library, providing identification of species and closely related taxa based on sequence similarity. The analysis takes approximately 1–2 h of preparation time with the major steps being DNA extraction, PCR amplification, and Sanger sequencing of the product, usually performed on a sequencing platform available in a core facility. Another method of species identification, chemotaxonomy, uses chemical profiles of biological markers, such as metabolites or surface molecules, as a chemical fingerprint. Chemical profiling as performed by DART MS is almost instantaneous, requires little or no sample preparation, and can provide preliminary structural identities of biomarkers particularly when paired with tandem MS.

**Figure 2:**
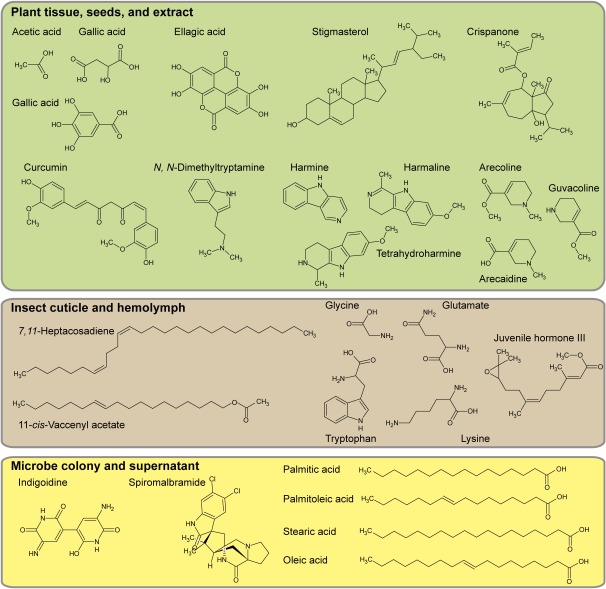
Fig. 2. Natural product chemicals detected by DART MS from plants, insects, and microbes. These compounds have been used as biological markers to separate closely related taxa, identify the provenance of foods and herbal medicines, and provide insight into fundamental aspects of physiology, behavior, or metabolism.

DART MS has been used to detect various classes of lipids as biomarkers of species and origin (Fig. 2). Antal *et al.* showed that sesquiterpene profiles could be used to discriminate between seeds of closely related herbs cumin, caraway, and fennel.^14)^ Similarly, red and white oak could be distinguished from each other by pyrolytic DART ionization of bark samples on the basis of short chain fatty acid (SCFA) profiles.^15)^ As a third example, Giffen *et al.* developed a high throughput method using DART MS to generate chemical fingerprints from *Salvia* (sage) leaves that allowed species differentiation based on signals corresponding to essential oil markers.^16)^ Intriguingly, DART MS profiling detected quantitative differences in profiles according to time of day (morning *vs.* evening) or age. Finally, plant membrane phytosterols, such as β-sitosterol and stigmasterol, have been used as markers for differentiating between pure and blended vegetable oils.^17)^ The presence of cholesterol in plant products can also serve as an indicator of adulteration from animal fat.^18)^

In addition to lipids, other chemical classes have proven to be useful analytical markers for DART MS chemical fingerprinting. DART MS together with TLC has been used to characterize turmeric-derived curcuminoids, the natural polyphenol compounds that are thought to be the active health-promoting compound in turmeric root^19)^ (Fig. 2). Surprisingly, despite its low mass range, DART MS is particularly advantageous for the analysis of large polysaccharides from plants, which range from tens to thousands of kDA. Conventional methods of analysis require chemical, physical, or enzymatic means to break down polysaccharides prior to chromatography-paired MS analysis. However, DART MS obviates a separate hydrolysis step since polysaccharides undergo thermal decomposition during DART ionization, generating characteristic profiles consisting of smaller fragments (*m*/*z* <350) in the ion source.^20)^ Many of the signals correspond to mono- and oligosaccharides. This feature was effectively used to characterize the origin of traditional Chinese herbal medicines (TCHM). TCHMs vary in quality and content due to the manufacturing process and provenance of ingredients. Ma *et al.* showed that six different herbal TCHMs could be distinguished on the basis of plant polysaccharide decomposition products.^20)^ Individual species also could be identified when mixed with one or two other species, indicating distinct polysaccharide compositions for each herb. Moreover, the same TCHM from different production regions also could be separated on the basis of DART ionization-produced polysaccharide fragments. In a second study, Zeng *et al.* applied DART MS to a popular TCHM injection, Danshen herb, and showed that products from different manufacturers could be classified on the basis of distinct salvianolic acid and saccharide profiles.^21)^ DART MS profiling could potentially serve as a facile method for the rapid fingerprinting and quality control of natural medicines.

### Profiling of plant mixtures

The analysis of complex plant mixtures is also amenable by DART MS. The chemical fingerprinting of propolis, a natural health supplement comprised of wax, bee saliva, and resinous plant exudates, revealed a series of signals corresponding to glycosides and phenolic compounds, amongst other small molecules. Using linear discriminant analysis, chemical profiles of propolis samples originating from various locations could be distinguished from each other.^22)^ As a second example, Lesiak and Musah showed that chemical fingerprints obtained from DART MS analysis of Ayahuasca brew were distinct for six different brew mixtures.^23)^ Whilst the psychoactive alkaloid *N*,*N*-dimethyltryptamine and β-carboline alkaloids harmine, harmaline, and tetrahydroharmine were present in all DART MS spectra (Fig. 2), the botanical profiles of each mixture could still be separated with the application of principal component analysis. In a related study, DART MS analysis of the mind-altering plant product Kanna revealed different alkaloid profiles depending on the commercial source and product variety.^24)^ Interestingly, dopants such as ephedrine were also identified in one of the blends, demonstrating the utility of DART MS profiling for detecting adulterants and counterfeits.

## INSECTS

### Direct analysis of cuticular lipids

The cuticular surfaces of insects are rich with lipids, amino acids, and other small molecules (Fig. 2). Some of these compounds function as pheromones, smell or taste signals that are sent and received by members of the same species and influence social behaviors such as mate finding, egg laying, and kin recognition.^25)^ The isolation, structural characterization, and functional assessment of insect pheromones have been areas of intense research in the field of natural product chemistry because of their importance to chemical ecology and application to pest control.^26)^ GCMS is the conventional method for measuring insect cuticular lipids, many of which are aliphatic saturated and unsaturated hydrocarbons. DART MS has been used increasingly for the classification of insects and characterization of pheromones due to its rapid analysis time and broader mass range. In one of the first applications to insect chemical ecology, DART MS was used to analyze CHCs from awake behaving *Drosophila melanogaster* (pomace fly).^10)^ Some CHCs function as pheromones and play pivotal roles in mate attraction and species recognition.^27)^ To examine how the CHC profile changes as a function of behavior, the cuticular profile of the same fly before and after mating was sampled with a nickel-plated brass sewing pin. DART MS analysis revealed several male-specific compounds on cuticles of recently mated females that were not present on virgin females. This finding indicates that males transfer multiple CHCs to females during copulation. Some of these molecules function as anti-aphrodisiacs that discourage mating from other males.^28,29)^

The ability of DART MS to expedite single insect profiling was effectively used in a genetic screen of intact organisms. The cuticular profiles of individual *Drosophila* from a library of 160 transgenic lines (5–10 biological replicates/line) were assayed by DART MS for defects in CHC production.^30)^ The screen identified 12 previously uncharacterized genetic pathways underlying cuticular hydrocarbon biosynthesis. In-depth molecular and genetic analysis of one of these pathways led to the characterization of a new ecdysone-related mechanism regulating pheromone-producing cells in *Drosophila*.

In addition to *Drosophila* studies, the broad mass range of compounds detectable by DART MS was a notable advantage in a recent work using DART ionization and laser desorption/ionization to profile *Nasonia* wasps. Both MS methods revealed the presence of very long chain CHC species (with carbon numbers from C25 to C52). Notably, many of the heavier compounds (above C41) were missed using GCMS. Principal component analysis indicated that CHC profiles were distinct for sex, age, and species.^31)^

### Applications to forensic entomology

**Figure 3:**
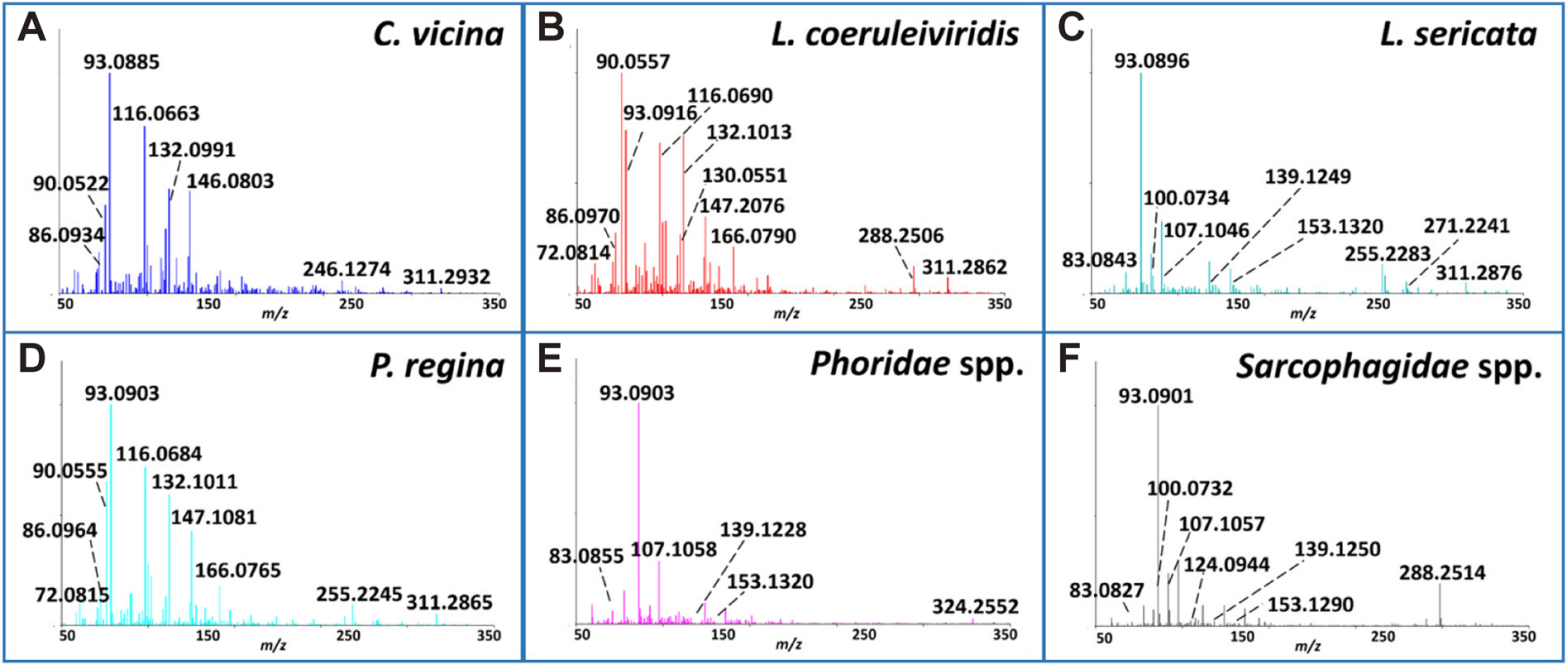
Fig. 3. DART MS profiling of ethanol suspensions containing eggs of necrophagous flies used in forensic analysis. (A–F) The following representatives from the *Calliphoridae* (blowfly), *Phoridae* (coffin fly), and *Sarcophagidae* (flesh fly) families were profiled: *Calliphora vicina*, *Lucilia coeruleiviridis*, *Lucilia sericata*, *Phormia regina*, *Phoridae* spp., and *Sarcophagidae* spp. Many of the signals have *m*/*z* consistent with amino acids including glycine (*m*/*z* 86), glutamic acid (*m*/*z* 148.08), phenylalanine (*m*/*z* 166), histidine (*m*/*z* 156), methionine (*m*/*z* 150), serine (*m*/*z* 106), threonine (*m*/*z* 120) and tyrosine (*m*/*z* 182). Signals corresponding to glutamine (*m*/*z* 147.08) and tryptophan (*m*/*z* 205) were detected only in *P. regina* ethanol suspensions. Figure reprinted with permission from https://pubs.acs.org/doi/10.1021/acs.analchem.7b01708^34)^ (further permissions related to the figure should be directed to the American Chemical Society).

Beyond pheromone analysis, DART MS is an effective means for classifying insect life stage, insect species, and insect populations. Chemical fingerprinting of insects is particularly useful for forensic entomology. One of the most popular and effective methods for determining time of death relies on the species and life stage of insect associated with corpses. Common carrion insects have predictable growth rates and appear within 5–15 min after death in a well-characterized sequence. The time of death estimates are largely based on the identity of blowfly (*Calliphoridae*) puparial or egg casings found on the body. However, identifying species based on the casing morphology is a time-intensive process that requires highly specialized knowledge. DART MS was successfully used to identify species based on fatty acid profiles of puparial casings^32)^ and egg-derived amino acids found in the ethanol solution in which the eggs are stored^33,34)^ (Fig. 3). Categorization of either type of chemical profile with linear discriminant analysis accurately distinguished between the different species. The analysis time, about 3 s per measurement, was a significant improvement over traditional methods of electron microscopy and DNA-based identification.

## MICROBES

### Chemical fingerprinting of bacteria and fungi

The development of rapid, accurate methods to identify microbial taxa has been an area of great interest because of its application to clinical microbiology and studies of the microbiome. Identifying microbial taxa based on metabolites or membrane profiles by MS is one promising approach that is already being used by clinical microbiology labs. Matrix-assisted laser desorption/ionization time-of-flight (MALDI-TOF)-based analytical platforms^35–37)^ obtain peptide mass fingerprinting (PMF) from pure bacterial cultures then match the profile against a proprietary reference database. Potentially, profiling with DART ionization could be an alternative approach for microbial chemotaxonomy. Cody *et al.* showed that DART MS fatty acid profiles distinguished between bacterial species.^38)^ Each of the 10 species were differentiated with 100% classification accuracy by principal component analysis based on the relative quantitative and qualitative differences in total fatty acid profiles. Notably, the bacteria used in the study belong to different genera. Further studies are needed to determine whether DART MS profiling is capable of differentiating between members of the same genus or subspecies. In addition to bacterial identification, DART MS could also be a promising method for fungal identification. Watts *et al.* showed that DART MS is capable of detecting secondary metabolites produced by *M. graminicola* fungal spores. Notably, the spores were sampled directly from the agar plate using an inoculation loop^39)^ rather than from a large volume of culture. The ease of sample preparation and sensitivity of DART MS make it an attractive complementary technology to MALDI-TOF PMF and genomic sequencing. Nonetheless, it will be critical to assess the efficacy of DART-based taxonomic identification when applied to samples containing a complex mixture of microbes.

### Single colony profiling

In addition to microbial chemotaxonomy, DART MS has also been used to profile metabolites within live bacteria colonies. *Leiseingera* is a bacterial symbiont of the Hawaiian bobtail squid.^11)^ The microbe is found in the jelly coating of squid eggs and produces protective antibacterial compounds against pathogens in the marine environment. DART MS was used to localize production of the putative antibacterial metabolite indigoidine directly from colonies of *Leiseingera* (Figs. 1B and 2). Different zones of a single colony, cultured either in the presence or absence of other bacterial species, were sampled with the tip of a sterile syringe needle that was subsequently analyzed by DART MS. When grown as a monoculture, the indigoidine signal was uniformly present throughout the *Leiseingera* colony. By contrast, when mixed with other bacterial strains, the metabolite signal was detected at highest abundance along the edges of the colony, possibly to facilitate antibacterial interactions. By using a probe-based sampling approach combined with DART MS, it was possible to analyze the same colony at two different time points, 1 and 7 d. The localization effect was more pronounced after 7 d of co-culturing with another species.

## METABOLITE ANALYSIS

The metabolomic content of biological fluids (*e.g.*, blood, urine, invertebrate hemolymph) reflects diet, drug intake, and environmental stress. Vitamins, lipids, amino acids, drug metabolites, and citric acid cycle intermediaries and products are amongst the types of molecules that are identified in metabolomic analyses. Bioinformatic tools provide preliminary identification of chemical species based on exact mass measurements and MS/MS data and can enable the reconstruction of biochemical pathways (see recent reviews^40,41)^). DART MS is well-suited for metabolomic analysis because of the relatively simple preparation, low memory effect between samples, and rapid analysis time. Several recent papers have optimized sample preparation and ionization parameters for DART MS to improve sensitivity, mass range, and reproducibility. Zhou *et al.* showed that derivatization of serum extracts with silylating reagents increased the number of detected signals by five-fold and broadened the mass range (up to *m*/*z* 800) compared to non-derivatized samples.^42)^ When paired with an automated sampling arm, technical replicates differed by 4.5% coefficient of variation (CV) comparing total ion chromatogram peak heights and 16.7–18.9% CV with respect to relative signal intensities.

DART MS application to metabolomic profiling has been shown with extracts from diverse sample types. DART MS analysis was used as a complement to NMR analysis of serum from whale sharks, confirming the presence of metabolites including amino acids, short chain hydroxy- and keto-acids, sugars, and osmolytes.^43)^ Notably, DART MS analysis of extracted serum revealed that 21 metabolites differed in relative abundance between healthy *vs.* unhealthy individuals. DART MS-based metabolomics also differentiated between muscle extracts of fish raised on different diets. Multivariate statistic distinguished between dietary treatments based on differences in triacyglycerols, organic acid, sugar, and fatty acid levels.^44)^

DART MS analysis has been used to quantify trace levels of hormones from insect hemolymph. Juvenile hormone III (JHIII; Fig. 2), a sesquiterpenoid, is an important developmental hormone in arthropods that has numerous physiological roles including the timing of development, regulation of other neuroendocrine molecules, and the production of eggs.^45,46)^ The high sensitivity of DART MS is well-suited for JHIII analysis and its precursors, all of which are found only in sub-picomole levels in the hemolymph.^47–49)^ With optimized helium gas flow rate and gas temperature, femtomole to sub-picomole amounts of synthetic standard was achieved.^50)^ When applied to hemolymph samples, DART MS analysis detected JHIII from *ca.* 0.5 μL of hemolymph (pooled from 50 individual flies) and showed that levels of JHIII in females increased after mating.^51)^

In addition to sera, DART MS-based metabolite studies have also been demonstrated with tissue samples. The metabolic products of plant use can be diagnostic for drug use. For instance, the intracellular uptake of areca alkaloids, metabolic products from areca nut (betel nut) chewing, may be useful as a biomarker for oral cancer risk.^52)^ Signals corresponding to the areca alkaloids and arecaidine/guvacoline (Fig. 2) were found with direct DART MS analysis of buccal cells scraped from the inner cheeks of areca nut chewers for up to 3 d post-chewing. DART MS has also been used to detect cocaine, amphetamine, 3,4-methylenedioxymethamphetamine^53)^ and tetrahydrocannabinol (THC)^54)^ from hair samples. In each of these examples, the tissues were measured directly in the ion source with no extraction step.

## CONCLUSION AND OUTLOOK

Since the introduction of DART MS ionization in 2005, the method has become well-established in the broad field of natural product chemistry. Natural product analysis is particularly challenging due to the ephemeral nature of the analytes and the complexity of the biological and chemical matrices in which they are found. The ability of DART MS to accommodate diverse sample types has resulted in a breadth of applications, from chemotaxonomy to behavioral studies. In addition, the relative ease with which DART ionization can be combined with orthogonal methods of separation, ionization, or online derivatization has expanded the breadth and depth of chemical information that can be obtained from a single analysis. One of the most exciting developments for DART MS is spatially resolved imaging. Recently, laser DART ionization (LADI) with a Nd : YAG laser (λ: 213 nm; fluence: 21 J cm^−2^; frequency: 20 Hz) was used to map the distribution of several alkaloids in *Datura leichhardtii* seeds with a resolution of *ca.* 110×50 μm.^2,55)^ Although the spatial resolution is still considerably poor compared to commercial MALDI imaging platforms, LADI does not require treating the substrate with matrix or solvent application.^55)^ It will be intriguing to see what else can be discovered from the natural world when DART ionization is paired with enhanced spatial capabilities.

## Notes

Mass Spectrom (Tokyo) 2019; 8(2): S0081
